# First Detection of *mcr-9* in a Multidrug-Resistant *Escherichia coli* of Animal Origin in Italy Is Not Related to Colistin Usage on a Pig Farm

**DOI:** 10.3390/antibiotics12040689

**Published:** 2023-04-01

**Authors:** Flavia Guarneri, Cristina Bertasio, Claudia Romeo, Nicoletta Formenti, Federico Scali, Giovanni Parisio, Sabrina Canziani, Chiara Boifava, Federica Guadagno, Maria Beatrice Boniotti, Giovanni Loris Alborali

**Affiliations:** Istituto Zooprofilattico Sperimentale della Lombardia e dell’Emilia Romagna—IZSLER, v. Bianchi 9, 25124 Brescia, Italy

**Keywords:** antimicrobial resistance, swine, critical antimicrobials, mobile colistin resistance, multidrug resistance, IncHI2 plasmid, *Enterobacteriales*

## Abstract

The emergence of colistin resistance raises growing concerns because of its use as a last-resort antimicrobial for the treatment of severe gram-negative bacterial infections in humans. Plasmid-borne mobile colistin resistance genes (*mcr*) are particularly worrisome due to their high propensity to spread. An *mcr-9*-positive *Escherichia coli* was isolated from a piglet in Italy, representing the first isolation of this gene from an *E. coli* of animal origin in the country. Whole genome sequencing (WGS) revealed that *mcr-9* was borne by an IncHI2 plasmid carrying several other resistance genes. The strain was indeed phenotypically resistant to six different antimicrobial classes, including 3rd and 4th generation cephalosporins. Despite the presence of *mcr-9*, the isolate was susceptible to colistin, probably because of a genetic background unfavourable to *mcr-9* expression. The lack of colistin resistance, coupled with the fact that the farm of origin had not used colistin in years, suggests that *mcr-9* in such a multidrug-resistant strain can be maintained thanks to the co-selection of neighbouring resistance genes, following usage of different antimicrobials. Our findings highlight how a comprehensive approach, integrating phenotypical testing, targeted PCR, WGS-based techniques, and information on antimicrobial usage is crucial to shed light on antimicrobial resistance.

## 1. Introduction

During the last decades, multidrug-resistant (MDR) bacteria have been emerging worldwide due to the misuse of antibiotics in human and veterinary medicine [1,2]. The spread of MDR bacteria is raising public health concerns globally and represents a growing challenge to healthcare systems [3]. In this scenario, preserving the efficacy of antimicrobials is paramount, especially those considered of critical importance for treating severe human infections. Colistin has been classified by the World Health Organisation as a highest priority critically important antimicrobial (HPCIA, [4]) as it is considered a last-resort treatment for severe infections caused by Gram-negative MDR bacteria in humans, especially in limited-resource settings [5,6]. However, the extensive use of this antimicrobial in livestock has led to the emergence of zoonotic colistin-resistant bacterial strains [7,8,9]. Colistin resistance in *Enterobacterales* can be either chromosomal- or plasmid-mediated [10,11]. Although chromosomal resistance has been known for a long time, mobile colistin resistance (*mcr*) genes were identified for the first time only in 2015 [12]. These genes are often plasmid-borne and have, therefore, a higher potential to spread globally through horizontal gene transfer [13,14]. Currently, ten different *mcr* genes have been reported worldwide and in many bacterial species, including *Escherichia coli*, *Enterobacter* spp., *Klebsiella* spp. and *Salmonella* spp., among others [15,16,17,18]. 

The emergence and spread of the MCR-family genes have led to policy changes worldwide, such as the ban of colistin use as a growth promoter in China in 2017 [19]. In the EU, where the usage of antimicrobials as growth promoters had been banned since 2006 [9], the marketing authorisations for oral veterinary medicinal products containing colistin combined with other antimicrobials were all revoked in 2016 [20]. Furthermore, the EU has also set targets to decrease colistin consumption in animals, leading to a 40% sales reduction between 2017 and 2021 [21]. In Italy, sales of colistin for veterinary use have declined steadily over the last 12 years, reaching a 98% reduction in 2021 [21], moving Italy from one of the highest to one of the lowest consumers in the EU. The first effects of this reduction policy already seem to be emerging; indeed, a drop in the circulation of *mcr*-1 genes was recently reported in Emilia-Romagna [22], an Italian region with a high pig density.

In 2022, as part of a larger project involving AMR surveillance, the colistin resistance gene *mcr-9* was identified in an *E. coli* isolated from a weaner pig with suspected enteritis. This strain was phenotypically and genetically tested for antimicrobial resistance; whole-genome sequencing (WGS) was performed and colistin usage in the farm was also investigated.

## 2. Results

The isolated *E. coli* strain (hereafter named E.coli_MDRbs22) belonged to the phylogenetic group B1 and tested positive to PCR for the resistance genes *bla*_CTX-M_ and *bla*_TEM_. The multiplex PCR tested negative for *mcr* genes 1 to 5 and positive for *mcr* 6 to 10 and particularly showed the presence of the *mcr-9* gene. Other samples from pigs with suspected enteritis were submitted by the same farm in 2022, and although some were positive for *E. coli*, including a few ESBL strains, no others carried the *mcr-9*. 

**Figure 1:**
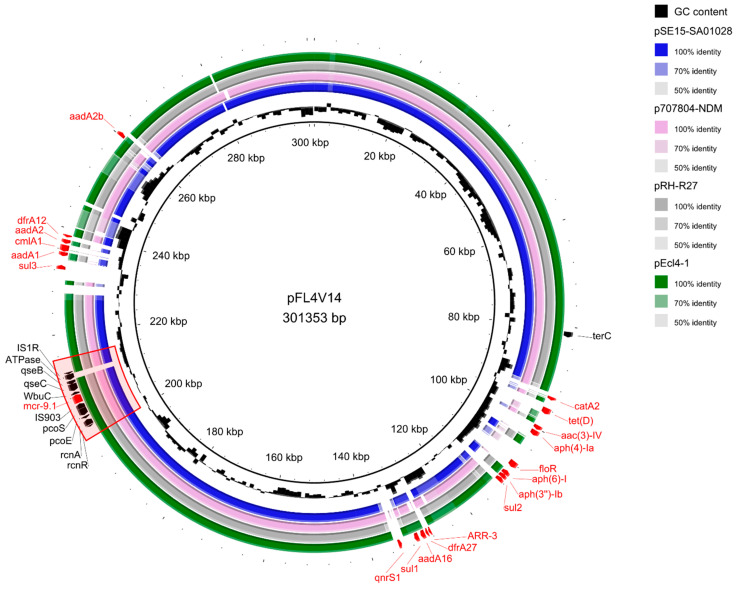
Circular map of IncHI2 plasmid pFL4V14 isolated from E.coli_MDRbs22 compared to the closest plasmids according to BLAST+ ([23], CP026661, LN555650, MH909331). Resistance genes and the *mcr-9* cassette have been highlighted in red.

The hybrid assembly of second- and third-generation sequencing data produced five large circular contigs: the chromosome (4,902,651 bp), an IncHI2/IncHI2A-type plasmid of 301,353 bp, an IncFII (pHN7A8) plasmid of 72,887 bp, a Col156 plasmid of 6723 bp and a plasmid of 6647 bp in length. The strain E.coli_MDRbs22 was identified as ST224 and typed as O91:H23. The mobile colistin resistance gene was identified as an *mcr-9.1* gene and was harboured on a conjugative lncHI2/IncHI2A-type plasmid of 301,353 kb in size, named “pFL4V14”. According to the IncHI2 pDLST scheme, this plasmid belonged to sequence type 1. In addition to *mcr-9.1*, several other resistance genes were found on this plasmid (identity: 100%, coverage: 99.65–100%): *ARR-3* (rifampicins resistance), *tet(D)* (tetracyclines resistance), *aadA1*, *aph(3‴)-Ib*, *aadA2b*, *aac(3)-IV*, *aph(4)-Ia*, *aadA2*, *aph(6)-Id*, *aadA16* (aminoglycosides resistance), *qnrS1* (quinolones resistance), *catA2*, *cmIa1*, *floR* (amphenicols resistance), *sul1*, *sul2*, *sul3* (sulphonamides resistance), *dfrA12*, *dfrA27* (trimethoprim resistance) (Figure 1). The pFL4V14 plasmid also carried the *terC* virulence gene (Figure 1), conferring resistance to tellurium and genes for silver, nickel, copper, arsenic and mercury tolerance. Additionally, resistance genes *bla*TEM-1B, *bla*TEM-214, *bla*TEM-206, *bla*TEM-141, *bla*CTX-M-55 (beta-lactams resistance) and rmtB (aminoglycosides resistance) were carried by the IncFII plasmid. 

When blasted against the NCBI database, pFL4V14 showed the highest coverage and identity scores (coverage > 86% and identity 99.99%) with some other IncHI2/2A plasmids harbouring *mcr-9.1*: pEcl4-1 (identity 99.99%, coverage 89%) found in *Enterobacter hormaechei* from a human clinical case in Canada (GenBank Accession Number: CP047741, [23]), pSE15-SA01028 (identity 99.99%, coverage 87%) in *Salmonella enterica* subsp. *enterica* from minced meat in Germany (CP026661), pRH-R27 (identity 99.99%, coverage 86%) in *Salmonella enterica* subsp. *enterica* from a pig farm in Germany (LN555650), and p707804-NDM (identity 99.99%, coverage 86%) found in a *Leclercia adecarboxylata* isolated in China (MH909331) (Figure 1).

**Figure 2:**
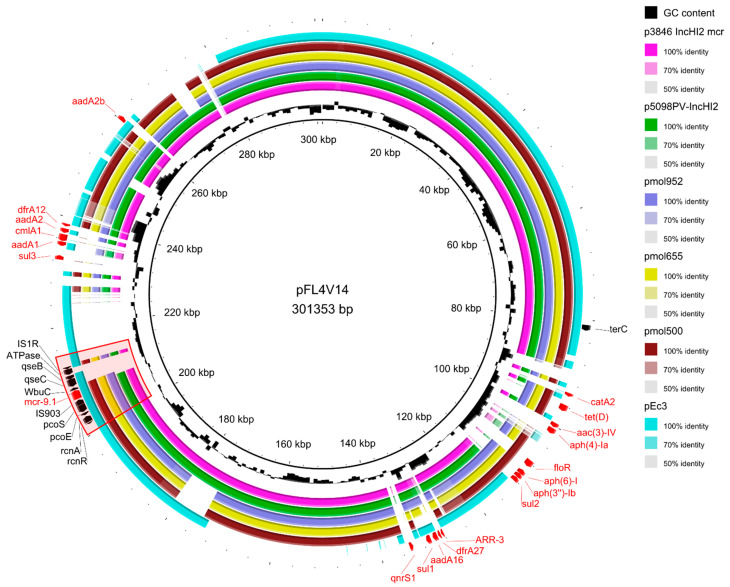
Circular map of IncHI2 plasmid pFL4V14 isolated from E.coli_MDRbs22 compared to other mcr-9-carrying plasmids found in Italy [24,25,26,27]. Resistance genes and the *mcr-9* cassette have been highlighted in red.

The comparison to previously identified *mcr-9*-carrying plasmids in Italy (Figure 2) revealed that our plasmid was relatively similar (99.96–99.98% identity and 80–82% coverage) to pMOL952, pMOL655, pMOL500 in *Salmonella enterica* subsp. *enterica* isolated in 2015–2019 from pigs and veal calves [24], to p3846_IncHI2_mcr in *E. cloacae* from a human [25] and to p5098PV from *Mixta calida* in a preterm newborn [26]. A high identity (99.99%) and a lower coverage (68%) were found by comparison with pEC3 in a multidrug-resistant human *E. coli* isolate [27].

The *mcr-9.1* gene was flanked upstream by an IS903 element and downstream by a wbuC family gene and the qseB/qseC regulatory system. The gene was included in the typical core structure of the *mcr-9* cassette composed of *rcnR-rcnA-pcoE-pcoS-IS903-mcr-9-wbuC*. Further downstream, our plasmid presented a gene coding an ATPase and an insertion sequence IS1R (Figure 1 and Figure 2). 

The strain was phenotypically resistant to 13 out of the 23 antimicrobials tested (corresponding to 6 different antimicrobial classes, Table 1); however, despite the presence of the *mcr-9.1*, it was susceptible to colistin (≤0.12 mg/L). 

## 3. Discussion

To the best of our knowledge, this is the first detection of the *mcr-9* gene in an *E. coli* of animal origin in Italy. The *E. coli* belonged to ST224, a ubiquitous genotype often associated with multiple resistances (in particular to carbapenems and other beta-lactams) and previously isolated in livestock (swine: [28,29]; poultry: [30]), as well as in humans [31,32], pets [33], wildlife [34] and water [35]). Indeed, our strain carried several resistance genes as well and was phenotypically resistant to multiple antimicrobial classes, although not to colistin, despite the presence of the *mcr* gene. Regarding the origin of the strain, the facts that this was an isolated finding and that the farm had been colistin-free for years (at least since 2019) suggest that E.coli_MDRbs22 originated in a different setting, entering the farm through purchased animals, contaminated well water or other external sources.

Together with *mcr-1*, *mcr-9* is the *mcr* gene the most widely disseminated worldwide, where it has been reported mainly in *Salmonella* spp. and *Enterobacter* spp. [36,37]. In Italy, plasmidic *mcr-9* has recently been detected in different bacterial species in samples of both animal and human origin [24,25,26,27], but never before in an *E. coli* of animal origin. Surprisingly, however, pFL4V14 was more similar to other plasmids sampled in Germany, Canada and China, again from both animals and human sources and in several bacterial species ([23]; CP026661, LN555650, MH909331), than to these plasmids isolated within the country.

The *mcr-9.1* in E.coli_MDRbs22 was carried by a large conjugative IncHI2/2A ST1 plasmid within an *mcr-9* cassette and was followed by the inducible regulatory system qseC/qseB. The genetic context of the gene was similar to that described in other IncHI2 plasmids in different Enterobacteriaceae, both in terms of the core structure of the cassette and in terms of downstream *WbuC* and *qseBC* genes [38]. Previous studies have shown that the presence of *mcr-9* is not always associated with phenotypical resistance to colistin (e.g., [14,39]). Although some authors found that *mcr-9* expression was inducible in the presence of colistin when located upstream of the two-component system *qseBC* [40,41], others highlighted that not all strains with *qseBC* were resistant to colistin [14], suggesting that the effectiveness of these regulatory systems in inducing *mcr-9* expression might be dependent on the genetic context and differ among strains. All the other resistance genes carried by E.coli_MDRbs22 conferred phenotypical resistance against the corresponding antimicrobial classes. For instance, the isolate also carried an IncFII plasmid carrying, in turn, the ESBL genes *bla*_CTX-M_ and *bla*_TEM_ and was indeed resistant to third- and fourth-generation cephalosporins. The strain, however, lacked *bla*_CMY_, which is associated with AmpC enzymes that are not inhibited by β-lactamase inhibitors [42,43,44], and was indeed susceptible to cefotaxime and ceftazidime combined with clavulanic acid. Most importantly, the IncHI2 plasmid carrying the *mcr-9.1* also harboured genes conferring resistance against amphenicols, aminoglycosides, quinolones/fluoroquinolones, sulphonamides, trimethoprim and tetracyclines. The presence of such genes, coupled with the lack of phenotypical resistance to colistin, suggests that the *mcr* may be the result of co-selection due to selective pressures on other resistance genes carried by pFL4V14. A similar conclusion was reached, for instance, by Mecesic et al. [45], who reported the presence of *mcr-9.1* in IncHI2 plasmids carrying multiple resistances in *Enterobacterales* isolated from human patients without any previous exposure to colistin. The frequent association of *mcr* with multiple resistance genes implies that they might spread silently and remain undetected for a long time, independently of colistin usage [26,40,45]. 

Our results confirm the importance of monitoring resistance genes even in contexts where a specific antimicrobial has not been administered in a long time, employing molecular methods to detect genes that may be silent in the carrier strain but that could be transferred to other bacteria, posing a risk to public health [46]. Considering that *mcr-9* has been widely reported in isolates of human origin, its spread could indeed represent a relevant issue for human medicine in the future [17,18,37]. A comprehensive approach, integrating traditional phenotypical testing, targeted PCR screening, WGS-based approaches and information on AMU is thus crucial to reduce the spread of AMR and to shed light on the genes responsible for it and on the mechanisms by which they affect bacterial sensitivity to the different antimicrobial classes. Finally, our findings further highlight that a One Health approach that includes farm monitoring is pivotal in tracking the spread of AMR genes in an effective manner. Currently, there is no surveillance system in Italy that integrates AMR data from human, livestock and environmental samples in a standardised manner. This represents a major public health gap that should be addressed in the future.

## 4. Materials and Methods

### 4.1. Farm Characteristics, Sampling and Isolation of the Bacterial Strain

An *E. coli* strain (E.coli_MDRbs22) was isolated as part of a routine diagnosis on a piglet with suspected enteritis and screened for extended-spectrum β-lactamase (ESBL/AmpC) and *mcr* genes within AMR monitoring activities. The pig had been reared in a farrow-to-feeder farm located in the north of Italy. The farm housed, on average, 880 sows, and the replacement gilts are always acquired from a single farm of the same owner. Pigs are fed with commercial feed, and water is supplied from a well.

According to the farmer and his veterinarian, neither of the two farms had used colistin since at least 2017. This statement could be confirmed, starting from June 2019, by consulting the Italian veterinary electronic prescription system implemented at a national level since that date. 

The microbiological diagnostic protocol included a pre-enrichment phase with 1 g of mesenteric lymph node placed in 9 mL of buffered peptone water and an overnight incubation at 37 °C. Then, a drop of broth was used to inoculate MacConkey agar supplemented with 1 mg/L Cefotaxime. The identification of phenotype-positive colonies was performed using the MALDI-TOF MS methodology. 

### 4.2. PCR Detection

A single colony from the phenotype-positive E.coli_MDRbs22 was sampled, and the DNA was extracted by lysis boiling (98 °C for 10 min). For the identification of *E. coli*, a PCR for phylogenetic group analysis was conducted according to Clermont et al. [47]. 

The detection of ESBL/AmpC and *mcr* resistance genes was performed through a panel of reactions. Specifically, multiple PCR was used to detect *bla*_CTX-M_, *bla*_SHV_, *bla*_TEM_ and *bla*_CMY_ using previously described primers [48,49]. PCR amplification reactions were performed in a volume of 25 µL containing: 1× of Buffer II (AmpliTaq Gold™ DNA Polymerase, Thermo Fisher Scientific, Waltham, MA, USA), 1.5 mM of MgCl_2_ 0.2 mM of dNTPs, 0.1 µM (*bla*_SHV_ and *bla*_TEM_) and 0.3 µM (*bla*_CTX-M_ and *bla*_CMY_) concentrations of each primer, 1 U/µL of enzyme AmpliTaq Gold™ DNA Polymerase and 5 µL of DNA template. The cycling parameters were as follows: an initial denaturation at 95 °C for 10 min; 30 cycles at 95 °C for 30 s, 59 °C for 45 s, and 72 °C for 60 s; a final extension at 72 °C for 5 min. The amplified PCR products were subjected to electrophoresis at a 2% agarose gel in 1× TBE buffer.

Two multiplex PCRs were set up using the QIAGEN Multiplex PCR Plus Kit for *mcr* genes from 1 to 5 and from 6 to 10 [50]. For the first nine *mcr,* we used primers described in [50,51], while for *mcr*-10 primers design was based on the first submitted gene sequence [52], as described in [16]. PCR reactions were composed as follows: *mcr 1–5* genes: 6.75 µL of RNase-free water, 12.5 µL of Multiplex PCR master mix (2×), 2.5 µL of Coral Dye and 1.25 µL of primer mix 10× (0.2 µL of each primer) *mcr 6–10* genes: 6.75 µL of RNase-free water, 12.5 µL of Multiplex PCR master mix (2×), 2.5 µL of Coral Dye and 1.25 µL of primer mix 10× (0.2 µL of each primer). 

### 4.3. Whole-Genome Sequencing and Bioinformatic Analyses 

We performed both short-read (on Miniseq, Illumina, San Diego, CA, USA) and long-read sequencing (on MinION, Oxford Nanopore Technologies, Oxford, UK). Genomic DNA extraction, Illumina libraries and sequencing runs on the MiniSeq platform were performed as previously described [16]. The DNA library for MinION was prepared using the Rapid Barcoding kit (SQK-RBK004) following the manufacturer’s instructions (www.nanoporetech.com, accessed on 2 March 2023). The sequencing was carried out through the R9-4.1 flow cell using 400 ng of DNA. The analysis required a 2 h run. Raw data collection and fast base-calling were performed using the software MinKNOW (v.22.08.4, Oxford Nanopore Technologies) and Guppy (v.6.2.7, Oxford Nanopore Technologies). The output was produced as *fastq* files with 1000 reads per file. After the quality control of the Illumina reads by FastQC v0.11.9 (www.bioinformatics.babraham.ac.uk/projects/fastqc/, accessed on 20 December 2022), and the cleaning with Trimmomatic v0.39 [53], a hybrid assembly of short Illumina reads and long MinION reads was carried out using Unicycler [54], with default parameters. Serotype, sequence type, virulence and antimicrobial resistance genes were identified using the following respective platforms: SerotypeFinder 2.0, Multi-Locus Sequence Typing (MLST 2.0), Virulence Finder 2.0 and ResFinder 4.1 tools (www.genomicepidemiology.org/services/, accessed on 28 December 2022).

The presence of the *mcr* gene in the obtained contigs was investigated using BLAST (blast.ncbi.nlm.nih.gov/Blast.cgi, accessed on 10 January 2023). The plasmid replicon and the plasmid type were identified using PlasmidFinder 2.0 and pMLST 2.0 (with IncHI2 DLST configuration) from CGE. The presence of conjugative regions in the plasmid was identified by oriTfinder (bioinfo-mml.sjtu.edu.cn/oriTfinder/, accessed on 10 January 2023; [55]). The sequence of the plasmid carrying *mcr-9* was queried against publicly available repositories (NCBI nucleotide collection—nr/nt, accessed on 16 November 2022) and compared with previously published plasmid sequences using BLAST+. Annotation was performed with Geneious Prime v2022.2.1 (www.geneious.com, accessed on 15 January 2023) using BLAST and RefSeq database (similarity cut-off: 94%), with manual curation of specific regions. Highly homologous complete plasmid sequences available in the NCBI database were compared with our plasmid using BRIG v0.95 (brig.sourceforge.net, accessed on 15 January 2023). Raw reads from both Illumina and Nanopore sequencing were submitted to the NCBI Sequence Read Archive (SRA) database under Project PRJNA939939.

### 4.4. Antimicrobial Susceptibility Testing

The isolate E.coli_MDRbs22 was subjected to antimicrobial susceptibility testing for the main antimicrobial classes through minimum inhibitory concentrations (MICs) technique (Sensititre Vizion Digital MIC, Thermo Fisher Scientific) using: (i) a commercial panel for colistin (Sensititre FRCOL plates, Trek diagnostics, Thermo Fisher Scientific), (ii) a commercial plate specific for surveillance on ESBL-producing isolates (Sensitre EUVSEC2 plates, Trek diagnostics, Thermo Fisher Scientific) and (iii) a customised in-house plate (Thermo Fisher Scientific), for a total of 23 antimicrobials tested. The strain was classified as susceptible or resistant based on epidemiological cut-off values (ECOFFs) recommended by the European Committee on Antimicrobial Susceptibility Testing (EUCAST, www.eucast.org last accessed on 2 March 2023). In the absence of ECOFFs, clinical breakpoints were used (CLSI, 2019; EUCAST, 2023).

## Figures and Tables

**Table 1:** Resistance genes detected by WGS in E.coli_MDRbs22 isolated from a pig, corresponding minimum inhibitory concentrations (MICs) and applied cut-offs for the tested antimicrobials. Resistances are highlighted in bold.

Antimicrobial Class	Resistance Genes	MIC		
		Antimicrobial	MIC Values (mg/L)	Cut-Off
Amphenicols	*catA2*, *cmIa1*, *floR*	Florfenicol	**>64**	>16 ^a^
Aminoglycosides	*aadA1*, *aph(3‴)-Ib*, *aadA2b*, *aac(3)-IV*, *aph(4)-Ia*, *aadA2*, *aph(6)-Id*, *aadA16*, *rmtB*	Aminosidine	>32	NA
		Gentamicin	**>32**	>2 ^a^
		Kanamycin	**>32**	>8 ^a^
Beta-lactams (Carbapenems)		Ertapenem	0.03	>0.03 ^a^
		Imipenem	≤0.12	>0.5 ^a^
		Meropenem	≤0.03	>0.06 ^a^
Beta-lactams (Cephalosporines and Penicillins)	*bla*_TEM-1B_, *bla*_TEM-214_, *bla*_TEM-206_, *bla*_TEM 141_, *bla*_CTX-M-55_	Cefazolin	**>8**	>4 ^a^
		Cefepime	**4**	>0.125 ^a^
		Cefotaxime	**64**	>0.25 ^a^
		Cefotaxime/clavulanic acid	≤0.06	>0.25 ^a^
		Cefoxitin	4	>16 ^a^
		Ceftazidime	**8**	>1 ^a^
		Ceftazidime/clavulanic acid	0.25	>1 ^a^
		Amoxicillin/clavulanic acid	8	>8 ^b^
		Ampicillin	**>32**	>8 ^a^
		Temocillin	8	>16 ^a^
Quinolones and Fluoroquinolones	*qnrS1*	Enrofloxacin	**>32**	>0.125 ^a^
		Flumequine	**>16**	>2 ^a^
Polymixins	*mcr-9*	Colistin	0.25	>2 ^a^
Sulfonamides and Diaminopyrimidines	*sul1*, *sul2*, *sul3* (sulfonamides); *dfrA12*, *dfrA27* (diaminopyrimidines)	Sulfisoxazole	**>512**	≥ 512 ^c^
		Trimethoprim/Sulfamethoxazole	**>16**	>0.5 ^a^
Tetracyclines	*tet(D)*	Tetracycline	**>16**	>8 ^a^

^a^ ECOFF www.eucast.org (last accessed on 2 March 2023). ^b^ The European Committee on Antimicrobial Susceptibility Testing (2023). Breakpoint Tables for Interpretation of MICs and Zone Diameters, Version 13.0. ^c^ Clinical and Laboratory Standards Institute (2019). Performance Standards for Antimicrobial Susceptibility Testing. 29th ed. Wayne, A: CLSI supplement M100.

## Data Availability

Sequencing data have been deposited open access in public databases. Other raw data supporting the conclusions of this article will be made available by the authors upon request, without undue reservation.
